# Euglycemic Diabetic Ketoacidosis in a Patient With Urinary Tract Infection

**DOI:** 10.7759/cureus.42594

**Published:** 2023-07-28

**Authors:** Sridhar Reddy Patlolla, Janaki Devara, Muhammad Atif Ameer, Prasanthy Reddy Patlolla, Madhusudhan Ponnala

**Affiliations:** 1 Hospital Medicine, Mission Hospital, Asheville, USA; 2 Internal Medicine, Mayo Clinic, Rochester, USA; 3 Medicine, Punjab Rangers Teaching Hospital, Lahore, PAK; 4 Internal Medicine, Suburban Community Hospital, East Norriton, USA; 5 Internal Medicine, Penn State Holy Spirit Hospital, Camp Hill, USA

**Keywords:** insulin requirement, type 1 and type 2 diabetes mellitus, endocrinology and diabetes, diabetic keto acidosis, euglycemia diabetic ketoacidosis, diabetes mellitis

## Abstract

Diabetes Mellitus (DM) is a complex metabolic disease primarily associated with elevated blood glucose levels in the body. Diabetic ketoacidosis (DKA) is the most feared acute presentation of diabetes mellitus (DM) in both type 1 and type 2 diabetes mellitus. Furthermore, euglycemic diabetic ketoacidosis (EDKA) is a relatively rare complication of DM in which the blood glucose levels are usually less than 250 mg/dl with an elevated anion gap metabolic acidosis. It can be a diagnostic challenge due to normal blood glucose levels and often can be overlooked. Physicians should be aware of EDKA; prompt diagnosis and treatment are critical in the timely management of the condition to prevent complications. We present a case of EDKA in a 74-year-old female precipitated by a urinary tract infection which was identified and treated promptly with insulin and dextrose infusion. In addition to that, an important difference between British and American guidelines has been highlighted.

## Introduction

According to the World Health Organization (WHO), the prevalence of diabetes is around 442 million patients globally [1], and there are 28.7 million known patients with diabetes in the United States of America (USA) according to the National Diabetes Statistics report by the Centers for Disease Control and Prevention (CDC) [2]. Diabetic ketoacidosis (DKA) is one of the most acute critical complications seen in patients with diabetes mellitus (DM), regardless of the type. Conventionally, DKA is associated with type 1 DM, but physiological stressors like infection and trauma can substantially increase the risk of DKA in other forms of DM. The overall incidence of DKA is less than four confirmed/probable cases per 1000 patient years [3]. DKA is a complex metabolic disorder secondary to absolute or relative insulin deficiency with various metabolic derangements. Prompt diagnosis and treatment decrease morbidity and mortality rates significantly.

Sodium-glucose cotransporter 2 (SGLT2) inhibitors are a class of oral anti-diabetics that work by acting on the proximal convoluted tubules and are notorious for precipitating DKA, hence should be discouraged in patients with the risk of euglycemic DKA (EDKA) [4]. Acute kidney injury in DKA is associated with increased morbidity and mortality and increased risk of chronic renal disease, especially in children and elderly patients who are already at risk for diabetic nephropathy [5]. The mortality rates for DKA are generally low, <1% in developed countries like the US and the UK, but the rates are substantially higher, up to 30% in developing countries. DKA is the leading cause of mortality in children and young adults with type 1 diabetes mellitus, accounting for up to 50% of mortality in this population [6]. Also, patients with COVID-19 take a prolonged time for the resolution of the DKA, and the mortality is much higher when compared with patients without COVID-19 infection [4-7]. In this case report, we present a classic case of EDKA and a review of the literature highlighting the important differences between the latest American and British guidelines in diagnosing and managing DKA.

## Case presentation

A 74-year-old woman with a history of type 2 diabetes mellitus presented with abdominal pain, nausea, and vomiting for 3 days prior to arrival. Her temperature was 98.2 F, her heart rate was 112 beats per minute, and her respiratory rate was 22 breaths per minute. Her home medication includes metformin 500 mg daily for her type 2 diabetes mellitus, and historically her blood glucose levels were well controlled. Reportedly, she has been noncompliant with her metformin for many days, and her oral intake has been poor for the last few days before the presentation.

On physical examination, the patient was ill-appearing and anxious, oriented to self only. Her BMI was 23.7 kg/m2. On auscultation, lungs were clear, and no murmurs, rubs, or gallops were heard; the abdomen was soft and non-tender, and on neurological examination, no sensory or motor deficits were noted except for orientation deficit, and cranial nerves II-XII were grossly intact. Her significant laboratory results are shown in Table 1. Her laboratory examination showed urinalysis positive for many bacteria, many white blood cells, nitrates positive, and ketones positive, with a specific gravity of 1.030. Her albumin level was 3.9 g/dl (normal range 3.6-5.1 g/dl). A computed tomography scan of the abdomen and pelvis showed diffuse thickening of the bladder wall, suggesting cystitis. The patient was diagnosed to have euglycemic DKA, likely triggered by sepsis secondary to her urinary tract infection. She was admitted to the intensive care unit (ICU) and treated with intravenous (IV) insulin per the latest DKA management guidelines and empiric ceftriaxone for urinary tract infection. Initially, we started her on a 10% dextrose IV infusion and insulin drip at the rate of 0.1 units/kg/hr. We did not give the bolus IV insulin. Her sugar levels dropped to less than 100 mg/dL within an hour, and we had to give 50 ml of dextrose 50% IV in addition to the ongoing dextrose 10% infusion, and the insulin rate was decreased to 0.05 units/kg/hr. The basic metabolic panel, magnesium, and phosphorous were monitored every 4 hours, and the electrolytes were repleted as needed. Within 24 hours, her anion gap was closed, kidney function was improving, her mental status was back to her baseline, and her diet was advanced after bridging with subcutaneous insulin. The patient was discharged home on day 3 of hospitalization on oral ciprofloxacin for the urinary tract infection.

**Table 1:** Significant Laboratory Results

Assay	Result	Reference Range & Units
Sodium	134	136 - 145 mmol/L
Potassium	5.4	3.5 - 5.3 mmol/L
Chloride	101	98 - 110 mmol/L
Bicarbonate	8.8	20.0 - 31.0 mmol/L
Blood urea nitrogen	70	6.0 - 24.0 mg/dL
Serum creatinine	4.5	0.5 - 1.0 mg/dL
Glucose	175	70 - 140 mg/dL
Anion gap	23.2	6 - 19 mmol/L
Serum osmolality	325	278 - 305 mOsm/kg
Beta hydroxy butyrate	3.59	0.00 - 0.30 mmol/L
Lactic acid	2.0	0 - 2 mmol/L
White blood cell count	14000	4500 - 11000
pH (Venous blood gas)	7.15	7.350 - 7.450
pCO2 (partial pressure of carbon dioxide)	25.4	32.0 - 45.0 mmHg
Hemoglobin A1C	5.8	4.0 - 5.6 %

## Discussion

DKA is a relatively uncommon clinical presentation of diabetes mellitus but is the most acute complication in people with diabetes mellitus, type I and II. It is a complex metabolic disorder that is secondary to absolute or relative insulin deficiency with a reflex increase in the counter-regulatory hormones (glucagon, cortisol, growth hormone, catecholamines) that will eventually result in hyperglycemia and ketosis. It is these high glucose levels and the metabolic acidosis from ketosis that are responsible for various metabolic derangements, like osmotic diuresis, electrolyte disorders, acute kidney injury, encephalopathy, etc., seen in DKA [8]. To make a diagnosis of DKA, all of these must be present: 1. Blood glucose concentration of more than 11 mmol/L or history of diabetes mellitus; 2. Blood ketone concentration of more than 3 mmol/L or significant ketonuria (2+ or more on standard urine stick); and 3. Bicarbonate concentration of less than 15 mmol/L and/or venous pH less than 7.3 [9].

According to this definition, patients with DKA can present with normal blood glucose levels, called euglycemic DKA. Hence all clinicians should be aware of this definition and should be prepared for early intervention and treatment of DKA.

The treatment of EDKA is no different from that of hyperglycemic DKA and consists of dextrose 10% fluid infusion (because the glucose is <14mmol/L) and IV insulin 0.1 units/kg/hr. If the glucose levels are still low while on dextrose 10% fluid, the insulin infusion rate should be decreased to half (0.05 units/kg/hr) to avoid hypoglycemia. The latest Joint British Diabetes Societies for Inpatient Care (JBDS-IP) 2023 guidelines recommend against using a bolus dose of insulin in the treatment of DKA. Subcutaneous long-acting insulin/analog should be continued while treating the DKA with rapid-acting IV insulin [10]. There is no such recommendation from the American Diabetes Association (ADA) [9-12].

Crystalloids rather than colloids are recommended fluids for volume resuscitation. The JBDS-IP 2023 recommends normal saline as the fluid of choice for resuscitation. Still, there are no studies favoring normal saline over balanced crystalloids like lactated Ringer's solution in the treatment of DKA [10]. In our practice, we avoid the use of normal saline and prefer lactated Ringer's solution because the chloride load in the normal saline can cause renal afferent arteriolar constriction, thereby reducing the renal blood flow and glomerular filtration rate, which can eventually lead to a worsening of the acute kidney injury and acidosis [13]. Balanced crystalloid solutions are increasingly recommended as first-line resuscitation fluids in patients with DKA, trauma, and patients undergoing surgery [14]. Studies have shown that treatment with balanced crystalloids, compared with normal saline, has resulted in rapid resolution of DKA, lesser hyperchloremia, and improved blood pressure profile and urine output. The rate of fluid administration has no difference in the outcomes. The rate of fluid infusion should be governed by the volume status and the hemodynamics in each case [15,16]. 

Routine administration of bicarbonate is no longer recommended. If pH > 6.9, no bicarbonate is needed. If the pH < 6.9, sodium bicarbonate is given until the venous pH is 7.0. It is recommended to infuse bicarbonate infusion every 2 hours if needed until pH reaches 7.0 [12]. With the widespread use of SGLT2 inhibitor class of drugs, the risk of developing euglycemic DKA is rising. This highlights the significance of using pH and ketones (rather than the older ‘glucose-centric’ care) to guide the diagnosis and management of DKA. Patients who develop DKA with SGLT2 inhibitors should stop using these agents immediately [10].

## Conclusions

DKA is a common medical emergency seen in patients with diabetes mellitus. Our patient was promptly diagnosed and treated with insulin and dextrose infusion without insulin bolus, resulting in a speedy recovery and successful outcome. Rapid diagnosis and treatment will decrease morbidity and mortality rates significantly. Physicians should be aware of the increasing incidence of euglycemic DKA, especially with the widespread use of SGLT2 inhibitors. So that if any patient with euglycemia comes in for an evaluation, the anion gap should be checked in order to timely diagnose the patient's condition. There is considerable overlap in the American and British guidelines, but there are important differences, as discussed above. Since diabetes mellitus is a common clinical entity virtually seen in any clinical practice, physicians should be aware of EDKA. Therefore, physicians should be aware of the latest updates in managing EDKA, and further large-scale studies/research are required to develop standard diagnostic and treatment protocols for EDKA across the globe.
